# Integrating clinical data into evidence-based practice: institutional guideline development for diabetic foot infections

**DOI:** 10.3389/fendo.2026.1752211

**Published:** 2026-02-09

**Authors:** Emmama Jamil, Muhammad Majid Aziz, Ummara Altaf, Waleed Mohammad Altowayan, Abdulmajeed Alqasoumi, Huda Arooj, Masaad Saeed Almutairi, Zikria Saleem

**Affiliations:** 1 Department of Pharmacy Practice, Faculty of Pharmacy, Bahauddin Zakariya University, Multan, Pakistan; 2 Department of Pharmacy Practice, Faculty of Pharmacy, Hamdard University Islamabad Campus, Islamabad, Pakistan; 3 Department of Environmental and Occupational Health, Tokyo University of Science, Tokyo, Japan; 4 Department of Pharmacy Practice, College of Pharmacy, Qassim University, Buraydah, Saudi Arabia

**Keywords:** antibiotic resistance, antibiotics, diabetic foot infections, empiric therapy, evidence-based practice, guideline development, public health

## Abstract

**Background/objectives:**

Diabetic foot infections (DFIs) are a major cause of morbidity in low- and middle-income countries such as Pakistan, where antimicrobial resistance (AMR) and the absence of localized guidelines complicate their management. This study aimed to analyze the microbiological profile of DFIs and develop evidence-based, context-specific empirical treatment recommendations.

**Methods:**

We conducted a retrospective study at a tertiary care hospital in Pakistan, reviewing all diabetic foot related admissions between January 2022 and December 2023. Patients with clinically suspected infections had wound cultures taken and were classified by infection severity using IDSA/IWGDF criteria. Wound specimens from 400 DFI patients were subjected to culture and CLSI-standard antimicrobial susceptibility testing. Pathogen patterns were analyzed across severity groups. A multidisciplinary stewardship team used these findings, together with international guidelines, to develop severity-specific institutional-based empiric treatment recommendations.

**Results:**

A total of 118 culture-positive samples from 400 admissions were analyzed. Gram-negative organisms predominated (62%), followed by *Escherichia coli* (25%), *Proteus mirabilis* (14%), and *Pseudomonas aeruginosa* (10%). *Methicillin-resistant Staphylococcus aureus* (*MRSA)* accounted for 23% and *methicillin-sensitive Staphylococcus aureus* (*MSSA)* 10%. *E. coli* was highly susceptible to tigecycline (86%) and amikacin (83%), while *Proteus mirabilis* was highly susceptible to carbapenems and piperacillin–tazobactam. *MRSA/MSSA* remain universally susceptible to vancomycin and linezolid. Based on these patterns, severity-stratified empiric regimens were recommended. Evidence grading supported strong recommendations for moderate to severe infections (SoR: Strong; QoE: Moderate).

**Conclusion:**

This study highlights the limited role of Access antibiotics in DFIs, with Watch agents serving as the main empiric therapy and Reserve drugs reserved for multidrug-resistant cases. Using hospital antibiograms, international standards, and expert consensus, we developed a locally tailored institutional guideline for DFI management, which is expected to enhance patient care and antimicrobial stewardship, with periodic updates required as resistance evolves.

## 1 Introduction

The prevalence of diabetes mellitus is increasing annually across the globe. According to the International Diabetes Federation (IDF) Diabetes Atlas 2025, approximately 589 million adults between 20–79 years of age are currently living with diabetes worldwide, representing nearly one in nine adults (11.1%) in this age group (1–3). As a result of multiple pathophysiologic changes, these patients commonly get affected by foot-related disorders, particularly infections, ulcers, and gangrene (4–6). Diabetic foot infections (DFIs) are among the most severe complications faced by individuals with diabetes mellitus, contributing significantly to morbidity, hospitalization rates, and healthcare costs globally (6–10). If not managed promptly, these ulcers can develop into soft tissue infection and osteomyelitis, dramatically increasing the risk of gangrene and limb amputation (11). The management of DFIs is complicated by the increasing prevalence of multidrug-resistant (MDR) organisms, which limits the use of narrow spectrum and cost-effective antibiotic choices and underscores the importance of culture-guided therapy (12).

International clinical practice guidelines, such as those developed by the Infectious Diseases Society of America (IDSA) and the International Working Group on the Diabetic Foot (IWGDF), provide structured, evidence-based approaches for the diagnosis and management of DFIs (13). These guidelines emphasize early recognition of infection, severity classification, and the use of empiric antimicrobial regimens tailored to common pathogens in high income countries (13–15).Furthermore, regional differences in antibiotic prescribing guidelines highlight the challenges of standardizing treatment approaches across different regions and have significant implications for future strategies to reduce antimicrobial resistance (AMR) (16).

In contrast, Pakistan, a low- and middle-income country (LMIC) with limited resources and diverse socioeconomic standards faces unique challenges in managing DFIs. The diabetes incidence in Pakistan is relatively high, so it ranks among the top 10 nations worldwide. Diabetes affected 5.2 million Pakistanis in 2000, but will likely affect approximately 13.9 million in 2030 (17). Despite efforts such as the establishment of the Pakistan Working Group on Diabetic Foot (PWGDF) and national programs to improve diabetic care, the absence of locally tailored, evidence based guidelines hinders effective infection control (18–20). As a result, empirical treatment often relies on clinician experience rather than microbiological evidence, increasing the risk of treatment failure and resistance development (21–23). In Pakistan, infections frequently involve MDR bacteria and access to advanced imaging or vascular interventions may be limited (24). With such a burden, evidence-based guidelines are essential for standardizing DFI management. The development of evidence-based, locally adapted treatment guidelines requires the integration of real-world clinical data and antimicrobial susceptibility patterns to support informed and context-specific decision-making. Therefore, it is important to integrate clinical data and local antimicrobial susceptibility patterns to formulate context-appropriate treatment recommendations.

The World Health Organization (WHO) introduced the Access, Watch, and Reserve (AWaRe) classification of antibiotics as a global antimicrobial stewardship framework to promote rational prescribing (25–27). The system classifies antibiotics as Access agents, which are narrow-spectrum first-line options with a low potential for resistance; Watch agents, which are broad-spectrum drugs with high resistance potential requiring careful monitoring; and Reserve agents, which are last treatment option for MDR infections and must be used under strict stewardship protocols (28–30). Although AWaRe provides a useful framework to optimize antibiotic use, its effective implementation in LMICs such as Pakistan is challenging because of distinct microbial landscapes and limited stewardship infrastructure (31–34). Therefore, integrating local microbiological data and antimicrobial susceptibility information into the AWaRe framework is essential to ensure context-appropriate guidelines.

Given the increased burden of DFIs and the high prevalence of AMR, there is an urgent need to develop evidence-based, locally adapted treatment guidelines for DFIs that incorporate real-world clinical data and antimicrobial sensitivity patterns. This study aims to address this gap by analyzing the demographic characteristics, microbial isolates, and antibiotic susceptibility profiles of patients with DFIs admitted to a tertiary care hospital in Pakistan. The findings are intended to inform context specific empirical antibiotic protocols that align with regional resistance trends and healthcare realities, ultimately supporting more effective and sustainable DFI management strategies. Adapting empirical treatment to reflect regional microbial trends and resistance profiles is essential for improving therapeutic outcomes, minimizing treatment failure, and promoting antimicrobial stewardship.

The aim of this study was to develop context-specific, evidence-based empirical treatment recommendations for diabetic foot infections (DFIs) by combining local microbiological data with established international guideline frameworks. Specifically, the study sought to describe the microbial profile of wound cultures obtained from DFI patients at our hospital, assess the antimicrobial susceptibility patterns of the isolated pathogens according to CLSI standards. Moreover, compare these local trends with recommendations from the IDSA/IWGDF guidelines and relevant global evidence. In addition, the study aimed to formulate severity-stratified empirical antibiotic recommendations (mild, moderate, and severe) using a structured multidisciplinary Antimicrobial Stewardship (AMS) approach and to grade these recommendations based on the Strength of Recommendation (SoR) and Quality of Evidence (QoE) criteria.

## 2 Methods

### 2.1 Study design

This study used a mixed methods design combined with a retrospective observational analysis of microbiological data with a structured antimicrobial-stewardship (AMS) guided guideline development process. The study was carried out at a tertiary care hospital in Pakistan and followed the STROBE recommendations for observational studies and the RIGHT statement with AGREE II considerations for clinical practice guideline development (35, 36). Antimicrobial susceptibility testing was carried in accordance with CLSI guidelines, and infection severity was classified using internationally validated criteria from the IDSA and IWGDF guidelines. These frameworks were used to support methodological rigor, and ensure reproducibility and comparability of the results. We retrospectively analyzed wound cultures from DFI patients admitted between January 2022 and December 2023.

#### 2.1.1 Patient selection

**Figure 1:**
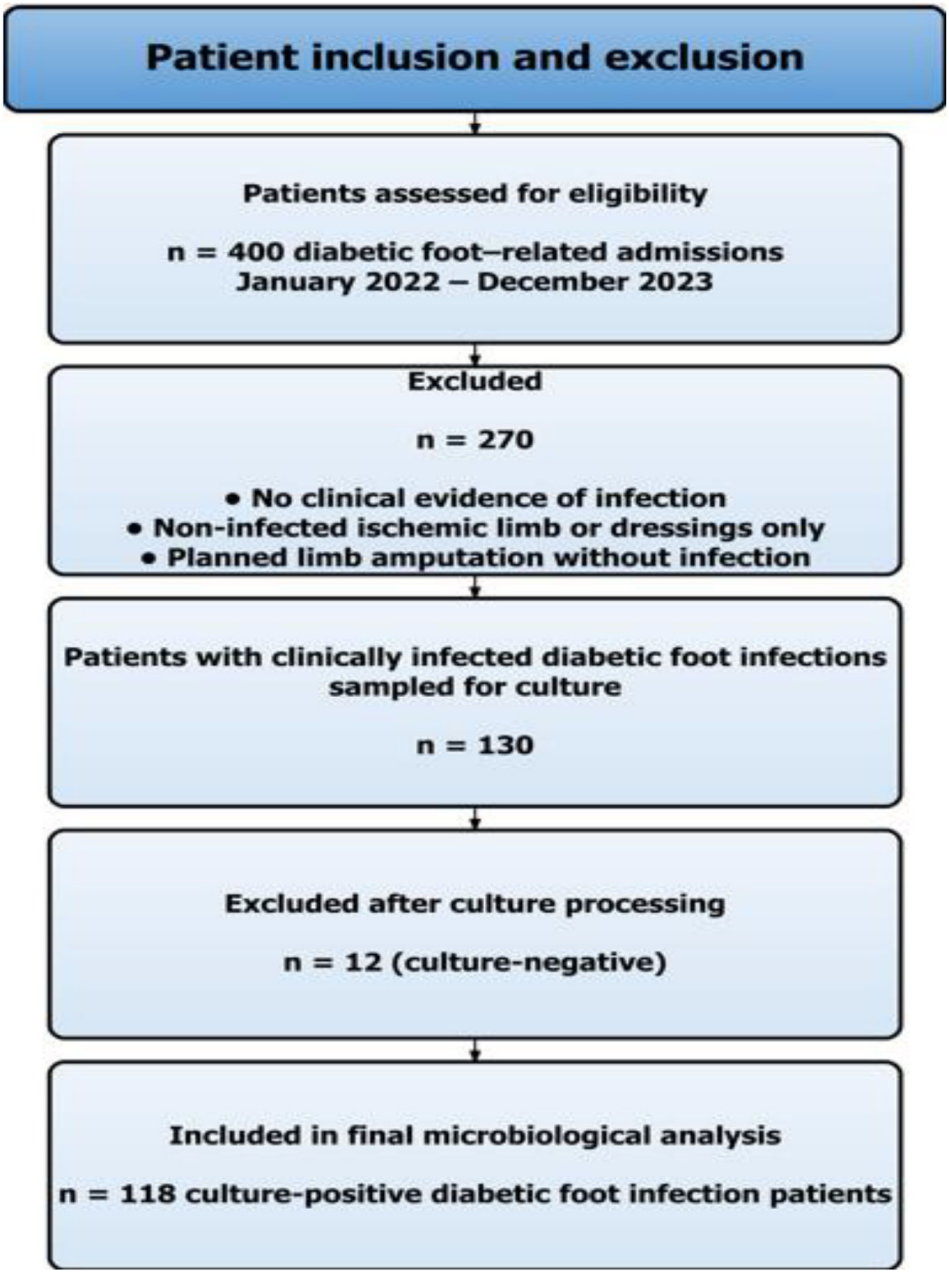
Flow diagram of patient inclusion and exclusion in the study.

All adult patients (≥18 years) included in the study had a confirmed diagnosis of DFI. The diagnostic criteria and severity classification followed the IDSA/IWGDF definitions (13). Patients inclusion and exclusion criteria are summarized in **Figure 1**.

Wound cultures were obtained only from clinically infected wounds, as per routine hospital practice. Patients admitted for amputation of non-infected ischemic limbs, dressings or unrelated surgical procedures were not sampled. Importantly, patients admitted solely for planned surgical amputation were not included because, antibiotic selection is not clinically relevant in such cases and cultures are not routinely obtained. This approach ensures that our microbiological data represent patients for whom empirical antibiotic therapy was clinically indicated. Prior antibiotic exposure was recorded when documented.

#### 2.1.2 DFI severity classification of infection

DFI severity was retrospectively assigned using IDSA/IWGDF criteria, based on detailed review of clinical notes, vital signs and wound assessments recorded at the time of culture selection. Severity was classified as:

Mild: Local infection of skin/subcutaneous tissue, if erythema present, >0.5 cm and ≤2 cm around the ulcer, no systemic signs.

Moderate: Erythema > 2cm or infection involving deeper tissues and < 2 signs of SIRS.

Severe: Local infection with ≥ 2 systemic inflammatory response syndrome (SIRS) criteria.

This classification guided both sampling decisions and subsequent interpretation of microbiological findings.

#### 2.1.3 Data collection

Upon admission, all DFI patients underwent a standardized clinical assessment. Patients’ past medical history was reviewed from hospital records, including documented comorbid conditions. As all included patients had diabetes mellitus, they were considered to have an underlying immunocompromised state. Other comorbidities were recorded when available; however, the study was not designed to stratify microbiological outcomes based on comorbidity or immune status.

Wound cultures were obtained only from clinically infected ulcers, prior to initiation of in-hospital antibiotics. Deep wound specimens (pus or wound aspirates) were collected; superficial wound swab cultures were avoided, and bone specimens were not routinely obtained. Information on prior antibiotic use before hospital presentation was inconsistently documented in the medical records. In the local context, antibiotics are widely accessible without prescription, making empirical community use common; therefore, many patients were likely to have received antibiotics before admission. Among the 130 cultured samples, 12 were culture-negative. Only the predominant pathogen per patient was included in the analysis; polymicrobial growth, when present, was uncommon and not analyzed separately. Specimens from infected wounds were collected following strict aseptic technique and immediately transferred to the microbiology laboratory to preserve diagnostic yield and minimize contamination. Samples were processed using routine aerobic microbiological culture techniques. Gram staining was performed as part of routine laboratory processing prior to culture. Specimens were inoculated onto standard culture media, including blood agar and chocolate agar, and pathogens were identified using conventional phenotypic methods. Antimicrobial susceptibility testing was performed using the disk diffusion method in accordance with CLSI guidelines (37).

Antimicrobial susceptibility testing (AST) covered a broad spectrum of agents commonly employed in the management of DFIs. These included penicillins and β-lactam/β-lactamase inhibitor combinations (e.g., ampicillin, amoxicillin–clavulanate, piperacillin–tazobactam), cephalosporins from first to fourth generation (cefazolin, ceftriaxone, ceftazidime, cefepime), and carbapenems (imipenem, meropenem, doripenem). Testing also encompassed fluoroquinolones (ciprofloxacin, levofloxacin), aminoglycosides (amikacin, gentamicin, tobramycin), and tetracyclines, including tigecycline. For Gram-positive organisms, glycopeptides (vancomycin, teicoplanin) and oxazolidinones (linezolid) were evaluated, while polymyxins (colistin, polymyxin B) were reserved for MDR Gram-negative infections.

For stewardship relevance, antimicrobials were also classified according to the WHO AWaRe framework (27, 38). Access group agents, recommended as first- or second-line therapy, included amoxicillin–clavulanate, first-generation cephalosporins, gentamicin, and ampicillin. Watch group agents, associated with higher resistance potential and intended for specific, limited indications, included third and fourth generation cephalosporins, fluoroquinolones, vancomycin, and carbapenems. Reserve group agents, considered last options for MDR organisms, included colistin, polymyxin B, linezolid, and tigecycline.

Demographics, clinical, laboratory data were recorded in standardized forms and entered into a secure electronic database for analysis. Non infected cases did not undergo cultures, ensuring that microbiological datasets represents only clinically infected DFIs in accordance with IDSA/IWGDF recommendations. Aggregated data were used to generate an antibiogram, which informed empiric therapy recommendations.

#### 2.1.4 Data analysis

Microbial distribution and antimicrobial susceptibility data were analyzed using descriptive statistical methods. Frequencies and proportions were calculated to summarize the prevalence of individual pathogens and the proportion of isolates susceptible to each antimicrobial agent. Data cleaning, tabulation, and visualization were performed using R software, which enabled structured interpretation of resistance patterns across Gram-positive and Gram-negative isolates.

#### 2.1.5 Guideline development

**Figure 2:**
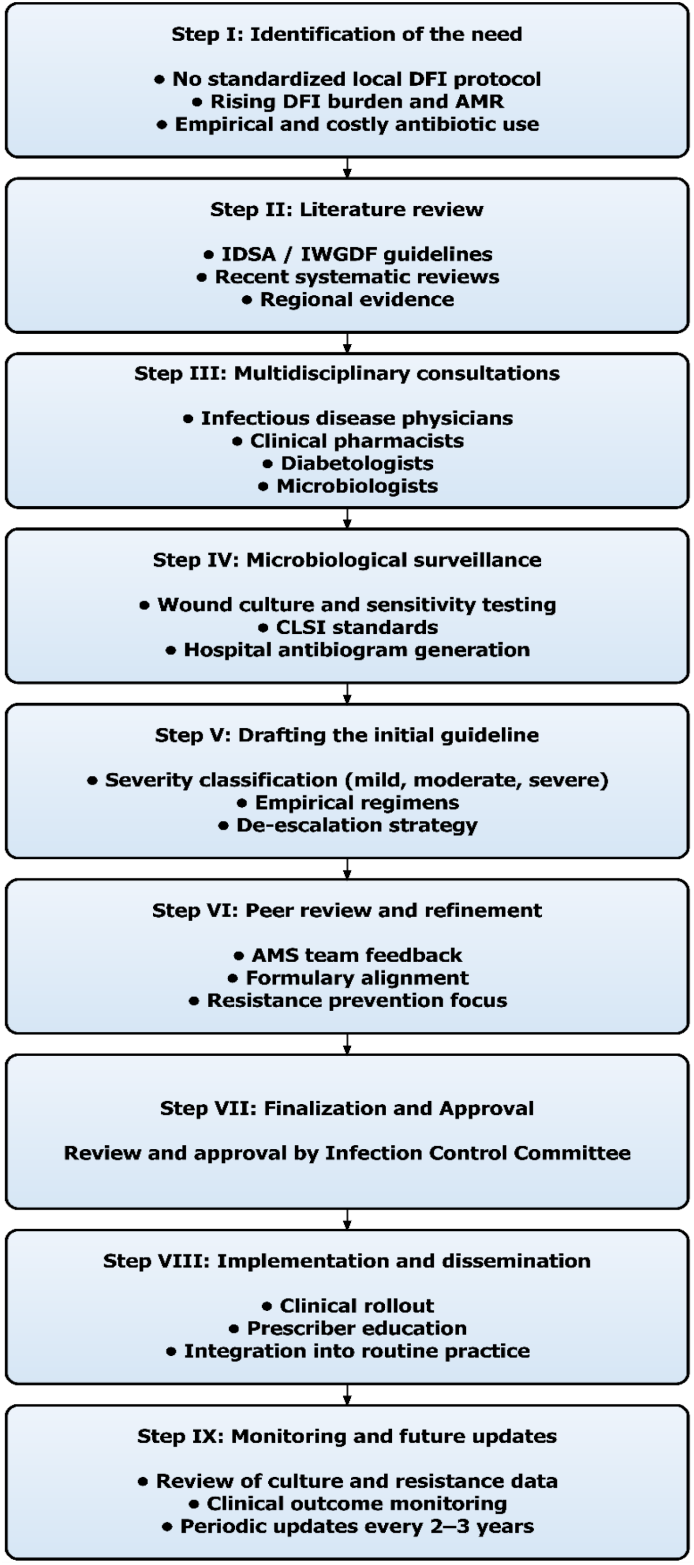
Stepwise framework for developing a context-specific antimicrobial guidelines for diabetic foot infections.

**Figure 2** summarizes the stepwise process followed for developing the context-specific antimicrobial guideline for diabetic foot infections.

#### 2.1.5.1 Step I: identification of the need

The need for guideline development was established during consultations with infectious diseases and surgical teams. No standardized local protocol for DFIs existed. Physicians relied on empirical prescribing, often broad-spectrum or costly, without reference to local resistance. With the rising DFI burden and high AMR prevalence, structured evidence-based guidance tailored to institutional needs were prioritized.

#### 2.1.5.2 Step II: literature review

A detailed literature review identified international standards for DFI management. The IDSA/IWGDF guidelines and institutional references were reviewed as gold standards (13). Recent systematic reviews and regional studies were also examined to ensure that the recommendations reflected current, relevant evidence.

#### 2.1.5.3 Step III: multidisciplinary consultations

This work was conducted by the hospital’s Antimicrobial Stewardship (AMS) Team, a multidisciplinary group responsible for optimizing antimicrobial use and infection management. The AMS team includes clinical infectious-disease physicians, clinical pharmacists, diabetologists, microbiologists, nursing staff, and health-information specialists. Pharmacists coordinated data extraction and literature synthesis, while clinical interpretation, validation of microbiological findings, and formulation of treatment recommendations were performed collaboratively with physicians and microbiologists. This ensured that all stages of the guideline-development process were clinically informed and multidisciplinary.

#### 2.1.5.4 Step IV: microbiological surveillance

Clinical specimens (pus/aspirates) were processed in the microbiology laboratory. The bacterial isolates were identified, and susceptibility testing was performed following CLSI protocols. Hospital antibiograms were generated to provide the microbiological basis for the guideline.

#### 2.1.5.5 Step V: drafting the initial guideline

The initial draft of the guideline was developed by integrating local resistance data, expert clinical judgment and international recommendations. It included diagnostic criteria, infection severity classification, and empirical regimens for mild, moderate, and severe infections. While awaiting microbiological culture and susceptibility results, the recommended antimicrobial regimens were suggested for initial empiric treatment in the inpatient hospital setting. Subsequent modification, de-escalation or switch to oral therapy was guided by microbiological findings and clinical response.

#### 2.1.5.6 Step VI: peer review and refinement

The draft was circulated for feedback and revised iteratively for clinical applicability. Emphasis was placed on aligning drug choices with the hospital formulary, ensuring efficacy while reducing unnecessary broad-spectrum use to support stewardship.

#### 2.1.5.7 Step VII: finalization and approval

The guideline was reviewed and approved by the hospital’s Infection Control Committee.

#### 2.1.5.8 Step VIII: implementation and dissemination

The guideline was disseminated via departmental meetings, workshops, and integration into prescribing protocols.

#### 2.1.5.9 Step IX: monitoring and future updates

A monitoring system was proposed with periodic review of culture data, clinical outcomes, and updates every 2–3 years, or sooner if major resistance shifts emerged.

### 2.2 Evidence grading

We adopted a pragmatic grading approach: Strength of Recommendation (SoR) was classified as “Strong” or ‘Conditional’, and “Quality of Evidence (QoE)” was classified as “High” (AST consistent with international guidance), “Moderate” (AST supported but with limited corroborating literature), or “Low” (expert consensus where data were sparse). Disagreements were resolved by consensus.

### 2.3 Ethics approval

Ethical approval for conducting the study was requested from the Human Ethical Committee of the Faculty of Pharmacy, Department of Pharmacy Practice, Bahauddin Zakariya University – Multan vide no.BZU-FOPDPP-2504.

As the study involved analysis of retrospective, fully anonymized hospital data collected during routine antimicrobial stewardship activities, the Ethics Committee granted a waiver of informed consent. All procedures complied with institutional and international standards for research involving human participants, and data confidentiality was strictly maintained throughout.

## 3 Results

Out of the 130 collected wound samples from hospitalized patients with DFIs, 118 (90.8%) yielded positive microbial cultures and were included in the final analysis. Among these patients, the majority were male (70%), while females comprised 30% of the cohort. Most individuals (87%) were adults aged under 65 years, and 13% were categorized as geriatric. Patient demographics and sample characteristics are summarized in **Table 1**.

**Table 1:** Demographic and sample characteristics of patients with diabetic foot infections.

Characteristic	N (%)
Gender	
Male	79 (70.0)
Female	39 (30.0)
Age group	
Adults	103 (87.0)
Geriatrics	15 (13.0)
Sample type	
Pus/aspirate	118 (100.0)
Microbes	
Gram negative	73 (62.0)
Gram positive	45 (38.0)
Total	118 (100.0)

In addition to demographic characteristics, the clinical spectrum of DFIs was also examined. The infections were classified into wound types; namely infected ulcers, abscesses, cellulitis, gangrene, and necrotizing fasciitis to assess both the severity and depth of infection. Infected ulcers were the most common, ranging from superficial to deep tissue involvement. Abscesses and cellulitis represented localized infections, whereas gangrene and necrotizing fasciitis reflected severe, limb-threatening conditions often associated with systemic signs. This classification not only reflected the clinical burden of infection but also provided the framework for interpreting microbiological findings and informing empirical treatment recommendations.

Microbiological analysis revealed a predominance of Gram-negative organisms, accounting for 62% of all the isolates, whereas Gram-positive organisms made up the remaining 38%. The most frequently isolated microorganism was *E.coli* (25%), followed closely by *methicillin-resistant Staphylococcus aureus* (MRSA) at 23%. Other common isolates included *Proteus mirabilis* (14%), *methicillin-sensitive Staphylococcus aureus* (MSSA) and *Pseudomonas aeruginosa* (10.3% each), *Streptococcus* sp*ecies* (5%), Enterobacter species (4.2%), *Klebsiella pneumoniae* and *Citrobacter* sp*ecies* (3.3% each), and *Acinetobacter baumannii* (1.6%). The frequency and distribution of microbial isolates from culture-positive DFI patients are presented in **Table 2**.

**Table 2:** Frequency and distribution of microbial isolates from culture-positive diabetic foot infection cases.

Microorganism	N (%)
*Escherichia coli*	30 (25.0)
*MRSA*	27 (23.0)
*Proteus mirabilis*	16 (14.0)
*MSSA*	12 (10.3)
*Pseudomonas aeruginosa*	12 (10.3)
*Streptococcus* sp*ecies*	6 (5.0)
*Enterobacter* sp*ecies*	5 (4.2)
*Klebsiella pneumoniae*	4 (3.3)
*Citrobacter* sp*ecies*	4 (3.3)
*Acinetobacter baumannii*	2 (1.6)
Total	118 (100.0)

Antibiotic susceptibility patterns vary across pathogens. *E. coli* exhibited the highest sensitivity to tigecycline (86%), amikacin (83%), and doripenem (79%), while demonstrating complete resistance to ampicillin and co-amoxiclav. Carbapenems such as imipenem, meropenem, and ertapenem showed relatively good efficacy (70–76%) against *E. coli*, although decreased sensitivity was noted in *Pseudomonas aeruginosa* (58–66%). Proteus mirabilis demonstrated excellent susceptibility to carbapenems (94–100%) and piperacillin-tazobactam (100%), but limited response to penicillins and certain cephalosporins. *Pseudomonas aeruginosa* showed moderate susceptibility to carbapenems and aminoglycosides, although resistance to third-generation cephalosporins and fluoroquinolones was evident.

Among the Gram-positive isolates, MRSA was highly susceptible to vancomycin, and linezolid. *MSSA* also showed high sensitivity to glycopeptides and oxazolidinones, in addition to aminoglycosides such as gentamicin (100%). Tetracyclines, particularly minocycline and tigecycline, showed good efficacy against both *MRSA* and *MSSA*. However, resistance to beta-lactams was common across Gram-positive isolates, notably with ampicillin and co-amoxiclav and this represents an alarming situation.

**Figure 3:**
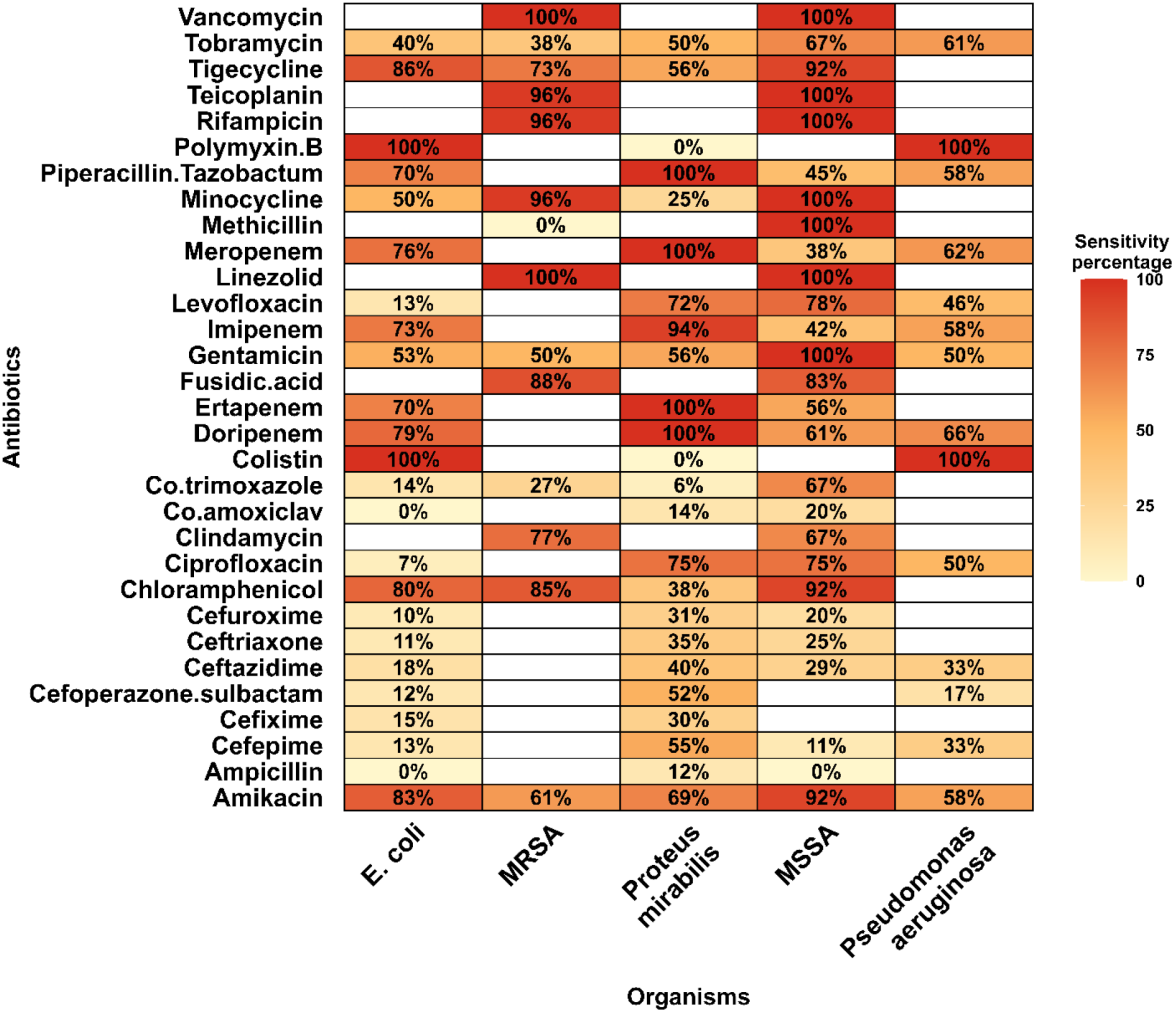
Heat map showing antimicrobial susceptibility patterns of major bacterial isolates causing diabetic foot infections. NT, Not tested Colors represent the percentage of susceptible isolates for each antibiotic–organism combination, with darker shades indicating higher susceptibility. Blank cells indicate antibiotics that were not tested (NT).

In terms of Class, penicillins and early-generation cephalosporins were largely ineffective against both Gram-positive and Gram-negative organisms. Carbapenems remained among the most potent options, although emerging resistance was noted in non-fermenting Gram-negative bacilli. Aminoglycosides and polymyxins demonstrated variable but generally acceptable sensitivity patterns, whereas glycopeptides and oxazolidinones were uniformly effective against Gram-positive organisms. Antimicrobial susceptibility patterns of major bacterial isolates are presented in **Table 3** and **Figure 3**.

**Table 3:** Antimicrobial susceptibility patterns of major bacterial isolates in diabetic foot infections.

Antibiotic	*Escherichia coli* (n = 30)	*MRSA* (n = 27)	*Proteus mirabilis* (n = 16)	*MSSA* (n = 12)	*Pseudomonas aeruginosa* (n = 12)
Penicillins					
Ampicillin	0 (0.0)	NT	2 (12.0)	0 (0.0)	NT
Co-amoxiclav	0 (0.0)	NT	2 (14.0)	2 (20.0)	NT
Piperacillin–tazobactam	21 (70.0)	NT	16 (100.0)	5 (45.0)	7 (58.0)
Cephalosporins					
Cefuroxime	3 (10.0)	NT	5 (31.0)	2 (20.0)	NT
Cefixime	4 (15.0)	NT	5 (30.0)	NT	NT
Cefoperazone–sulbactam	4 (12.0)	NT	8 (52.0)	NT	2 (17.0)
Ceftazidime	5 (18.0)	NT	6 (40.0)	3 (29.0)	4 (33.0)
Ceftriaxone	3 (11.0)	NT	6 (35.0)	3 (25.0)	NT
Cefepime	4 (13.0)	NT	9 (55.0)	1 (11.0)	4 (33.0)
Carbapenems					
Imipenem	22 (73.0)	NT	15 (94.0)	5 (42.0)	7 (58.0)
Meropenem	23 (76)	NT	16 (100.0)	5 (38.0)	7 (62.0)
Ertapenem	21 (70.0)	NT	16 (100.0)	7 (56.0)	NT
Doripenem	24 (79.0)	NT	16 (100.0)	7 (61.0)	8 (66.0)
Fluoroquinolones					
Ciprofloxacin	2 (7.0)	NT	12 (75.0)	9 (75.0)	6 (50.0)
Levofloxacin	4 (13.3)	NT	12 (72.0)	9 (78.0)	6 (46.0)
Aminoglycosides					
Amikacin	25 (83.0)	16 (61.0)	11 (69)	11 (92.0)	7 (58.0)
Gentamicin	16 (53.0)	14 (50.0)	9 (56.0)	12 (100.0)	6 (50.0)
Tobramycin	12 (40.0)	10 (38.0)	8 (50.0)	8 (67.0)	7 (61.0)
Tetracyclines					
Minocycline	15 (50.0)	26 (96.0)	4 (25.0)	12 (100.0)	NT
Tigecycline	26 (86.0)	20 (73.0)	9 (56.0)	11 (92.0)	NT
Glycopeptides/Oxazolidinones					
Methicillin	NT	0 (0.0)	NT	12 (100.0)	NT
Linezolid	NT	27 (100.0)	NT	12 (100.0)	NT
Vancomycin	NT	27 (100.0)	NT	12 (100.0)	NT
Teicoplanin	NT	26 (96.0)	NT	12 (100.0)	NT
Polymyxins					
Polymyxin B	30 (100.0)	NT	0 (0.0)	NT	12 (100.0)
Colistin	30 (100.0)	NT	0 (0.0)	NT	12 (100.0)
Others					
Clindamycin	NT	21 (77.0)	NT	8 (67.0)	NT
Fusidic acid	NT	24 (88.0)	NT	10 (83.0)	NT
Co-trimoxazole	4 (14.0)	7 (27.0)	1 (6.0)	8 (67.0)	NT
Rifampicin	NT	26 (96.0)	NT	12 (100.0)	NT
Chloramphenicol	24 (80.0)	23 (85.0)	6 (38.0)	11 (92.0)	NT
Total	30 (100.0)	27 (100.0)	16 (100.0)	12 (100.0)	12 (100.0)

Antimicrobial susceptibility patterns stratified by WHO AWaRe classification are summarized in **Table 4**. Access agents showed limited effectiveness, Watch group drugs provided the mainstay for moderate to severe infections, whereas Reserve agents retained excellent activity but were restricted for MDR cases in line with stewardship principles.

**Table 4:** Antimicrobial susceptibility of major DFI pathogens, organized by WHO AWaRe Classification.

AWaRe group	Antimicrobial agents	Key findings	Implications for guideline
Access	Amoxicillin–clavulanate, ampicillin, 1st–2nd gen cephalosporins (cefazolin, cefuroxime), gentamicin, co-trimoxazole	Poor activity of ampicillin and early cephalosporins against Gram-negatives; Amoxicillin–clavulanate retained moderate activity in mild DFI (esp. streptococci, MSSA); Gentamicin provided partial Gram-negative coverage	Limited role for empiric use beyond mild DFI; may be used for targeted therapy once cultures are available
Watch	3rd–4th gen cephalosporins (ceftriaxone, cefotaxime, ceftazidime, cefepime), Fluroqinolones (ciprofloxacin, levofloxacin), carbapenems (imipenem, meropenem, doripenem), vancomycin, teicoplanin	Gram-negative resistance to fluoroquinolones was high; E. coli retained low susceptibility to cefepime/ceftazidime; Proteus mirabilis highly susceptible to carbapenems and piperacillin–tazobactam; *MRSA/MSSA* fully susceptible to vancomycin and teicoplanin	Remains mainstay for moderate DFIs; vancomycin essential for MRSA; carbapenems should be reserved for severe infections or ESBL risk
Reserve	Tigecycline, linezolid, colistin/polymyxin B	E. coli highly susceptible to tigecycline (86%); MRSA/MSSA universally susceptible to linezolid; Polymyxins preserved for carbapenem-resistant Gram-negatives (rarely required in DFI)	Reserve agents highly effective but should be restricted to severe or MDR cases; emphasized in guideline for stewardship

On the basis of local resistance profiles and infection severity classifications (mild, moderate, and severe), empirical antibiotic guidelines were formulated to reflect the regional microbial landscape, current susceptibility trends, and antibiotic availability within the healthcare setting. The framework was designed to support clinicians in selecting appropriate empirical therapies while awaiting definitive culture and sensitivity results, thereby promoting timely, effective, and rational antibiotic use in DFI management. For mild infections, co-amoxiclav combined with clindamycin was recommended, with ciprofloxacin or fusidic acid as alternatives. For moderate infections, vancomycin plus piperacillin and azobactam was suggested as the first-line option, with linezolid, clindamycin, or fluoroquinolones considered suitable alternatives. For severe infections with systemic involvement, vancomycin in combination with carbapenems or piperacillin–tazobactam was preferred, whereas alternative regimens including clindamycin, aminoglycosides, or fluoroquinolones were selected according to renal function and local resistance patterns. Severity-stratified empirical antibiotic regimens recommended for DFIs are presented in **Table 5**.

**Table 5:** Recommended severity specific empirical antibiotic regimens for diabetic foot infections in the context of regional resistance trends.

Disease severity	Preferred choice	Alternative choice	Comments
Diabetic foot ulcer			
Mild (skin and subcutaneous tissue only). If erythema, must be > 0.5 cm to ≤ 2 cm around the ulcer.	Co-amoxiclav + clindamycin	Ciprofloxacin + clindamycin/fusidic acid	Final regimen based on CS report. Duration of treatment depends on the presence of osteomyelitis and clinical response.
Moderate (local infection with erythema > 2 cm, infection involving deeper tissues, and < 2 signs of SIRS).	Vancomycin + piperacillin–tazobactam	Linezolid + clindamycin + ciprofloxacin/levofloxacin	Monitor RFTs and observe the patient for pseudomembranous colitis.
Severe (local infection with ≥ 2 signs of SIRS).	Vancomycin + piperacillin–tazobactam or carbapenems (imipenem, meropenem, doripenem)	Vancomycin + clindamycin + aminoglycosides or ciprofloxacin/levofloxacin	Monitor renal function tests and observe the patient for pseudomembranous colitis.

NB: RFTs, Renal Function Tests; SIRS, Systemic Inflammatory Response Syndrome; C/S, Culture and Sensitivity.

## 4 Discussion

This study provides critical observations regarding the microbiological profile and antibiotic resistance patterns associated with DFIs in a tertiary care setting in Pakistan. These findings highlight the predominance of Gram-negative organisms, particularly *E. coli*, which contrasts with many Western studies where Gram-positive organisms, especially *S. aureus*, tend to dominate. However, our results are consistent with those of regional studies in South Asia, where Gram-negative pathogens are increasingly involved in DFIs due to environmental exposure, poor wound hygiene, and widespread antibiotic misuse (39). Gram-negative bacteria account for 76.27% of the isolates from Pakistani DFIs, with *E. coli* (15.72%), *P. aeruginosa*, and *P. mirabilis* as key pathogens (40, 41). This contrasts markedly with Western studies, where Gram-positive bacteria such as *S. aureus* dominated 40-60% of cases (42).Although *E.Coli* and *MRSA* were among the most frequent pathogens overall, the majority of mild- severity infections in our study were superficial ulcers in which Gram-positive cocci particularly streptococci and MSSA remained the predominant organisms. Accordingly, our empiric recommendations for mild DFIs prioritize narrow spectrum agents targeting typical Gram positive pathogens, while broader Gram negative coverage is reserved for moderate and severe infections. This stratified approach is consistent with IDSA/IWGDF principles and aligns with antimicrobial stewardship goals by minimizing unnecessary exposure to broad spectrum antibiotics in clinically mild presentations.

The high prevalence of multidrug-resistant organisms, including *MRSA* and *ESBL* producing *Enterobacteriaceae*, reflects a serious threat to empirical treatment strategies (43–46). The degree of resistance to commonly used antibiotics such as ampicillin, co-amoxiclav, and cephalosporins is alarmingly high, indicating that traditional empirical regimens may no longer be effective in this setting. The relatively preserved activity of carbapenems against *E. coli* and *P. mirabilis*, along with the efficacy of amikacin and tigecycline, underscore the need to reserve these agents for confirmed or highly suspected resistant infections, especially given concerns around toxicity, cost, and the risk of promoting further resistance (20, 24, 47, 48).

Importantly, glycopeptides and oxazolidinones maintained strong efficacy against *MRSA* and *MSSA* isolates, supporting their continued use in severe infections caused by Gram-positive pathogens. Nonetheless, the moderate resistance of *MRSA* to agents such as clindamycin and co-trimoxazole reinforces the importance of culture-directed therapy. These findings also underscore a significant loss of susceptibility to narrow-spectrum and cost-effective antimicrobial agents (48).The sustained efficacy of glycopeptides such as vancomycin and oxazolidinones such as linezolid against *MRSA* and *MSSA* isolates in DFIs highlights their critical role in managing severe Gram-positive infections, particularly in regions with high *MRSA* prevalence. Vancomycin remains universally effective against *MRSA*, despite emerging concerns about increasing minimum inhibitory concentrations (MICs), whereas linezolid offers strong clinical efficacy and oral bioavailability, making it valuable for outpatient care (49, 50).This approach not only optimizes treatment success but also helps preserve last-line antibiotics such as carbapenems and polymyxins for confirmed resistant infections, mitigating further resistance development.

These findings also have significant implications in the context of the WHO AWaRe classification. In our study, many of the antibiotics that demonstrated the highest activity such as carbapenems, colistin, and glycopeptideswere included within the Watch and Reserve groups. This means that Access antibiotics, which the WHO recommends as first-line therapy, are largely ineffective in our setting. The reliance on Watch and Reserve antibiotics for empirical therapy undermines global stewardship goals and highlights the urgent need for region-specific AWaRe adaptations. The incorporation of local antibiogram data into the AWaRe framework may help balance stewardship principles with the clinical imperative of ensuring effective empirical coverage in resource-constrained healthcare systems.

Our findings highlight the urgent need for local antibiograms to guide empirical therapy and highlight the limitations of applying international guidelines such as those from the IDSA or IWGDF without contextual adaptation. For example, broad-spectrum cephalosporins commonly recommended in international protocols have shown limited effectiveness in our patient population, further justifying the creation of locally tailored empirical guidelines (51–53). Moreover, The preserved efficacy of colistin in DFIs offers a crucial last-line defense against MDR Gram-negative pathogens such as *Pseudomonas aeruginosa*, particularly in regions with high carbapenem resistance. However, its use is fraught with significant challenges, including a well-documented toxicity profile-ranging from nephrotoxicity in 10–50% of patients to neurotoxicity which complicates its administration in diabetic populations that are often burdened by preexisting renal impairment (54).

Therefore, the development of empirical treatment protocols based on the basis of local sensitivity patterns stratified by infection severity represents a practical step toward improving treatment outcomes. These guidelines, if adopted and regularly updated, can serve as a cornerstone for antimicrobial stewardship programs, particularly in resource-limited settings where culture facilities and infectious disease expertise are often scarce.

### 4.1 Study limitations

The guideline development process faced various practical challenges. Because this was a single-center study, the findings may not be fully applicable to other healthcare settings with different patient populations, prescribing practices, or antimicrobial resistance trends. However, the primary goal of this study was to develop an institution-specific, evidence-based guideline based on local microbiological data. These findings provide a foundational framework that can support future multicenter surveillance and the development of locally adapted antimicrobial stewardship initiatives. A major limitation was the restricted availability of microbiological data. Cultures were obtained from only 130 of the total 400 admitted patients with diabetic foot related conditions, of which 118 yielded positive results. This reflects routine clinical practice, where cultures are collected only from infected wounds; while non-infected ischemic ulcers and patients admitted for amputations are not regularly sampled. Prior antibiotic use before hospital admission may also have contributed to culture negative results. As a result, the final microbiological dataset represents the subsets of patients for whom empirical antibiotic treatment was clinically required, which limits broader generalization of microbial trends across all DFI patients.

Due to laboratory capacity constraints, anaerobic cultures were not performed, which may have led to underestimation of anaerobic pathogens, especially in deep or ischemic DFIs. This reflects the diagnostic limitations of many resource limited hospitals in LMICs. Furthermore, antibiotics are widely accessible without prescription in Pakistan, making pre-hospital antimicrobial use common. Despite using standard techniques, our ability to evaluate the impact of prior antibiotic use on microbiological yield was further limited by incomplete documentation of specific agents used and duration of treatment.

While the sample size limits inferential generalizability, it adequately captured the dominant bacterial spectrum and resistance profiles that inform empiric prescribing. Future multicentre or prospective surveillance will further validate these findings.

## 5 Conclusion

This study systematically integrated international evidence, local microbiological surveillance, and multidisciplinary expertise to develop an evidence-based antimicrobial guideline for diabetic foot infections. The findings demonstrated that Access group antibiotics have limited empirical utility in this setting, while Watch group agents remain the cornerstone for moderate to severe infections, and Reserve agents retain high efficacy but must be preserved for MDR cases. By generating hospital-wide antibiograms and translating them into context-specific treatment recommendations, this work addresses a critical gap in standardized local practice. The resulting guideline promotes rational antibiotic use, supports antimicrobial stewardship, and is expected to improve both clinical outcomes and resource utilization. Periodic updates will be essential to align with evolving resistance trends and ensure sustained clinical relevance.

## Data Availability

The original contributions presented in the study are included in the article/supplementary material. Further inquiries can be directed to the corresponding author.
